# Effectiveness of YouTube as a Source of Medical Information on Heart Transplantation

**DOI:** 10.2196/ijmr.2669

**Published:** 2013-11-21

**Authors:** He-Ming Chen, Zhong-Kai Hu, Xiao-Lin Zheng, Zhao-Shun Yuan, Zhao-Bin Xu, Ling-Qing Yuan, Vinicio A De Jesus Perez, Ke Yuan, Mark Orcholski, Xiao-Bo Liao

**Affiliations:** ^1^Division of Cardiothoracic SurgeryThe Second Xiangya HospitalCentral South UniversityChangshaChina; ^2^Complex Carbohydrate Research CenterUniversity of GeorgiaAthens, GAUnited States; ^3^College of Computer ScienceZhejiang UniversityHangzhouChina; ^4^Division of Medical InformaticsStanford University Medical CenterStanford, CAUnited States; ^5^Institute of Metabolism and EndocrinologyThe Second Xiangya HospitalCentral South UniversityChangshaChina; ^6^Division of Pulmonary and Critical Care MedicineStanford University Medical CenterStanford, CAUnited States

**Keywords:** heart transplantation, Internet, medical informatics, online videos, YouTube, e-learning

## Abstract

**Background:**

In this digital era, there is a growing tendency to use the popular Internet site YouTube as a new electronic-learning (e-learning) means for continuing medical education. Heart transplantation (HTx) remains the most viable option for patients with end-stage heart failure or severe coronary artery disease. There are plenty of freely accessible YouTube videos providing medical information about HTx.

**Objective:**

The aim of the present study is to determine the effectiveness of YouTube as an e-learning source on HTx.

**Methods:**

In order to carry out this study, YouTube was searched for videos uploaded containing surgical-related information using the four keywords: (1) “heart transplantation”, (2) “cardiac transplantation”, (3) “heart transplantation operation”, and (4) “cardiac transplantation operation”. Only videos in English (with comments or subtitles in English language) were included. Two experienced cardiac surgeons watched each video (N=1800) and classified them as useful, misleading, or recipients videos based on the HTx-relevant information. The kappa statistic was used to measure interobserver variability. Data was analyzed according to six types of YouTube characteristics including “total viewership”, “duration”, “source”, “days since upload”, “scores” given by the viewers, and specialized information contents of the videos.

**Results:**

A total of 342/1800 (19.00%) videos had relevant information about HTx. Of these 342 videos, 215 (62.8%) videos had useful information about specialized knowledge, 7/342 (2.0%) were found to be misleading, and 120/342 (35.1%) only concerned recipients’ individual issues. Useful videos had 56.09% of total viewership share (2,175,845/3,878,890), whereas misleading had 35.47% (1,375,673/3,878,890). Independent user channel videos accounted for a smaller proportion (19% in total numbers) but might have a wider impact on Web viewers, with the highest mean views/day (mean 39, SD 107) among four kinds of channels to distribute HTx-related information.

**Conclusions:**

YouTube videos on HTx benefit medical professionals by providing a substantial amount of information. However, it is a time-consuming course to find high-quality videos. More authoritative videos by trusted sources should be posted for dissemination of reliable information. With an improvement of ranking system and content providers in future, YouTube, as a freely accessible outlet, will help to meet the huge informational needs of medical staffs and promote medical education on HTx.

## Introduction

Heart transplantation (HTx) is still the gold standard in the treatment of end-stage heart failure for appropriate candidates [1]. Since the first successful human-to-human HTx was performed in 1967, the survival quality and life span of HTx recipients have improved tremendously [2]. Between 1982 and 2009, there were 97,911 cases of HTx in the world, according to the Registry report of the International Society for Heart and Lung Transplantation (ISHLT) [3]. Along with the evolving development in patient selection, surgical techniques, perioperative care, and clinical follow-up, the outcomes of HTx have improved over the past four decades [1]. The overall survival rates after HTx show inspiring results from the data of ISHLT: five-year overall survival rates were 62.49-68.94%, ten-year 47.53-52.08%, and fifteen-year 29.63-37.05% [4]. Furthermore, the quality of life of HTx recipients is excellent. For instance, if including housewife recipients, approximately 90% of the adult recipients returned to their job following HTx in Japan [5]. Nevertheless, the volumes of HTx recently slowly declined, largely due to a critical organ donor shortage, and there were approximately 2200 cases yearly in the United States [6,7]. Therefore, the shortage of live surgical cases has led to reduced opportunities to witness this major operation, especially for medical students and trainee doctors.

At present, the Internet has become the largest and most up-to-date source for medical information worldwide [8]. In North America alone, 74% of adults use the Internet daily, and 80% of all users search for health-related information [9-11]. Acquiring and sharing medical information via the Internet offers extraordinary electronic-learning (e-learning) possibilities and has gradually changed the learning habits of medical professionals. When questions about health care arise, physicians increasingly turn to the Internet, which has changed the way medical students learn, communicate, and share specialized information, rather than to journals and textbooks [12,13]. Major search engines, such as Google, are often the first place physicians go for information [13-15]. Since 2005, YouTube has become the third most visited site on the Internet, after Facebook and Google [9,16]. Presently, there are over 4 billion hours of video being watched per month on YouTube, with 72 hours of video uploaded per minute, triple the statistical outcome in 2010 [17,18]. Therefore, the YouTube website is currently the leading audiovisual information center of medically relevant videos [13]. Numerous individuals, organizations, hospitals, and academic institutions from around the world have uploaded plenty of freely accessible medical videos onto the YouTube website. Moreover, the new generation of medical professionals is inclined to use social networks, online communities, and multiple media to learn specialized knowledge, because these means possess a nature of immediacy and parallelism when presenting information [19]. Currently, 94% of medical students actively participate in social media applications, compared to 79% of residents and 42% of physicians [19,20].

Some scholars have evaluated YouTube as a source of medical information on H1N1 influenza, papillomavirus vaccination, prostate cancer, and kidney stone [21-24]. However, until now, little is known about the characteristics of existing YouTube videos focusing on HTx. To our knowledge, there is no investigation to have examined the quality of videos related to HTx on YouTube. In this study, our aim is to assess the overall situation of specialized medical information in HTx-related YouTube videos.

## Methods

### Determination of HTx-Related Videos

**Figure 1:**
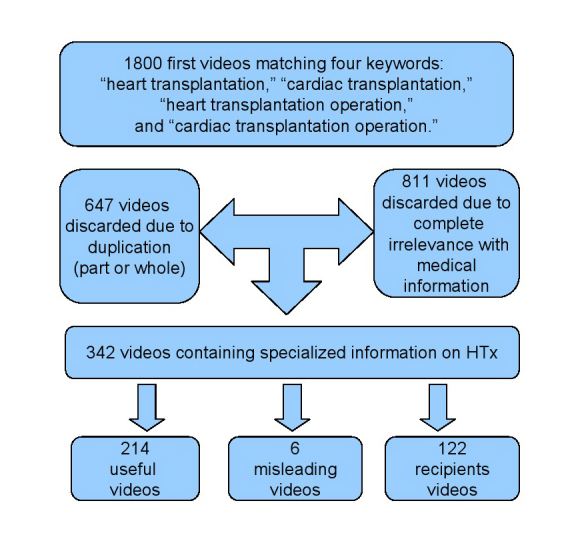
A graphic display of the classifying process of HTx-related videos.

This trial was conducted as a cross-sectional analysis. The website YouTube (YouTube, LLC, San Bruno, CA) was searched according to “relevance” priority for the following keywords: “heart transplantation”, “cardiac transplantation”, “heart transplantation operation”, and “cardiac transplantation operation.” All the videos containing relevant information about HTx before February 01, 2013 were included in this study. The total number of videos that appeared in the searching was 6930. However, 95% of people conducting an online search will watch no further than the first 60 videos of output, and most researchers for similar studies on YouTube videos usually chose the first 200 to 300 videos as their data sources [24-26]. Thus, we viewed and analyzed the first 1800/6930 (25.97%) videos (600 in 3750 “heart transplantation”, 600 in 1200 “cardiac transplantation”, 300 in 770 “heart transplantation operation”, and 300 in 1210 “cardiac transplantation operation”), on the assumption that no medical practitioner would go beyond the first 300 to 600 videos even for a serious e-learning goal. English language (comments or subtitles) in the video was a prerequisite for inclusion. Among all the videos, those meeting this inclusive criterion were further viewed. Data evaluation was independently conducted by two experienced cardiac surgeons (H-M Chen and X-B Liao) blind to each other. After discarding all the videos that were either duplicated, which have partially or completely identical content with shorter durations (part or whole, 647/1800, 35.94%), or completely irrelevant with medical knowledge (811/1800, 45.05% such as the names of some songs or electronic games), all the videos containing specialized medical information on HTx (342/1800, 19.00%, such as surgical lectures or live broadcasts) were classified from the aspect of information and knowledge as useful, misleading, and recipients videos (Figure 1). This classifying methodology of our study was conducted in accordance with the observations of Sood et al [24,27,28].

First, the video was grouped under the “useful” category if it contained scientifically correct information about any aspect of HTx such as donor procurement, surgical techniques, perioperative management, rejection control, clinical follow-up, new technologies, or the issues about medical humanities (eg, history of HTx, appealing for organ donation, etc). Second, the video was categorized as “misleading” if it contained scientifically incorrect or unproven information for now (eg, self-healing from serious heart failure by the help of the God rather than medical service; asserting that mechanical circulatory support is currently a practical alternative to HTx, but without convincing proof existing in the literature; oversight during the delivery and preservation of cardiac graft; and dissemination for abandoning HTx in end-stage heart failure patients). Finally, if the video described recipients’ personal experience rather than medical information of HTx per se (eg, for raising donation, expressing gratitude, advertising hospitals, etc), it was called a “recipients video.”

In case the investigators’ designation was not identical, the video was reevaluated with both surgeons and a united assessment was conducted. The kappa statistic was used to measure interinvestigator variability.

### Basic Communicative Analyses of HTx-Related Videos

Videos were categorized according to the “source” into four groups: namely, hospital/university channel (H/UC), independent user channel (IUC), medical dotcom channel (MDC), and news agency channel (NAC) [21,24]. These four categories were determined after watching all the videos and were chosen on the basis of the primary themes that emerged. Data were analyzed according to six kinds of YouTube characteristics that include (1) “total viewership”, (2) “duration”, (3) “source”, (4) “days since upload”, (5) “scores” given by the viewers, and (6) specialized information contents of the videos. To begin with the evaluation process, first, the “scores” of videos were used to describe the general evaluation of each video, which were determined by subtracting the total number of “dislikes” from the total number of “likes” designated by antecedent viewers at the statistical point in time. In our opinion, the higher positive value of “scores” may mean higher recognition of antecedent viewers and more attractive to new viewers.

Data were analyzed with SPSS version 19.0 (SPSS Inc) and presented as mean (SD). When more than two groups of means were compared (eg, mean “duration”, mean number of “days since upload”, and mean “scores” for four source groups), data were analyzed for statistical significance using a one-way analysis of variance (ANOVA) followed by a Tukey comparison of all groups. Before running the analysis, data were checked to determine if they met the assumptions of ANOVA (homogeneity of variances and data were sampled from the Gaussian distribution). *P* value less than .05 was considered statistically significant.

## Results

### Top 10 YouTube Videos After Searching With the Term “Heart Transplantation”

After the first-step search with four key terms, the vast majority of the output consisted of nonmedical or minute-medical videos without meaningful and specialized information (such as live but shaking or silent videos of less than 10 seconds). For example, a YouTube search for the term “heart transplantation” returned 3750 videos, and the top 10 outcomes sorted by relevance are provided in Table 1. Observably, the first four videos and the last three videos are useful for medical professionals. However, three intermediate videos (ranked 5th, 6th, and 7th) are all the advertisements of an electronic game named “Surgeon Simulator 2013.” In short, 3 days after uploading, the number of their “total viewership” accounted for 291,760/1,069,235 (27.28%) of the number of whole “total viewership” of these 10 videos. Furthermore, the “scores” of these 3 videos, which are completely uncorrelated with HTx, unexpectedly showed high value (1003, 394, and 2469, respectively).

### Classification of HTx-Related Videos

Of all the YouTube videos that were viewed, 342/1800 (19.00%) videos were classified into three groups containing medical information on HTx (total duration, 2373.8 minutes). Details for the inclusion of these videos are shown in Figure 1. The kappa coefficient of agreement on classification of videos between two surgeons was .89, which is usually in the “almost perfect” agreement range (.81-.99) [39]. The classification of these HTx-relevant videos based on their usefulness with details of other characteristics is given in Table 2. The mean duration of 342 videos was 6.94 minutes (SD 11.59, range 0.5-93), the mean scores of them showed 18.77 (SD 99.69, range 42 to 1148), and the mean viewers/day (since the date videos being uploaded)” were 15.95 (SD 67.14, range 0.02-785.30).

The majority of the useful videos were mainly posted by strong reputation such as H/UC (88/214, 41.1%) and NAC (64/214, 29.9%) (Table 2). For example, the “batsonhospital” channel provided the third ranked video in Table 1, which documented a success story about the fourth pediatric HTx in the Batson Children’s Hospital at the University of Mississippi Medical Center (see Multimedia Appendix 1). On the contrary, IUC delivered a majority of the misleading videos (5/6, 83%) and recipients videos (45/126, 35.7%). Detailed analysis of useful videos is shown in Table 3. Videos uploaded by H/UC occupied the vast proportion both in numbers (88/214, 41.1%) and total duration in minutes (657.05/1723.7, 38.12%) among all videos. No statistically significant difference was noted in the mean “duration” (*P*=.55), mean “days since upload” (*P*=.25), and mean “scores” (*P*=.28) among useful videos based on “source” by ANOVA. However, useful IUC (40/214, 18.7%) videos had significantly higher mean “viewers/day” than H/UC videos (*P*=.006) and NAC videos (*P*=.046).

### Communicative Analyses of Useful and Misleading Videos

**Figure 2:**
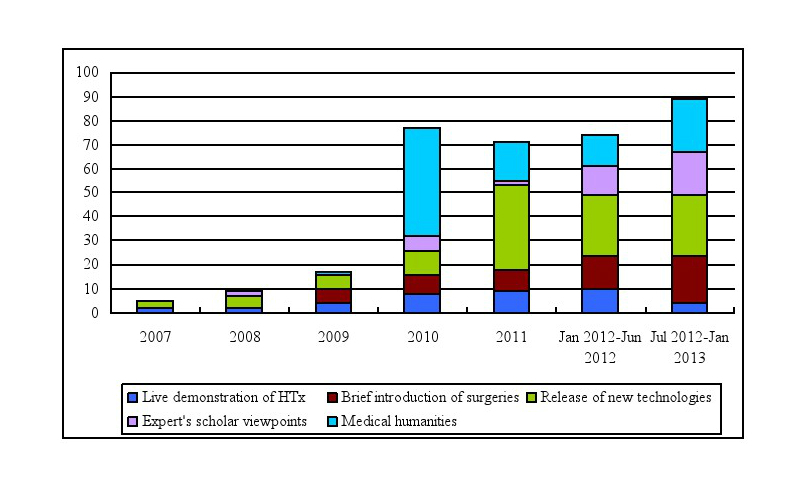
The annual numbers of useful HTx-related videos.

Useful videos were also analyzed based on the medical information they delivered. All useful videos contained HTx-related information on at least one or more of the following aspects: (1) live demonstration of HTx, (2) brief introduction of surgeries, (3) release of new technologies, (4) scholar viewpoints by experts, or (5) medical humanities. For example, videos were categorized as “medical humanities” if the main message of the videos portrayed the history of HTx or brainstormed on the issue of organ donation after brain death. Content analysis of useful videos with respect to the above five aspects is presented in Table 4. The annual number of useful HTx-related videos shows an uptrend since 2007, especially with a growth spurt in 2012 (Figure 2).

Compared with useful videos, the misleading videos demonstrated the following characteristic: fewer numbers, shorter total and mean “duration”, but higher mean “scores” and mean “viewers/day” (Table 2). These results suggest that the misleading videos might have a more influence on audience compared to useful videos. However, because the data of misleading videos presented a non-Gaussian distribution with a small number of samples (just 6 videos), we did not further analyze these data.

**Table 1:** A summary of the top ten videos ranked by their “relevance”^a^ resulting from a YouTube search for “heart transplantation” (HTx) on February 01, 2013.

Rank	Video title	Number of viewers	Days since upload	Scores	Description^b^	Reference
1	Heart transplantation	141,443	384	393	Showing orthotopic “bicaval” technique	[29]
2	Heart transplant surgery	188,083	898	307	Showing a brief scene of HTx	[30]
3	Revived Heart Transplant Program at Batson Children’s Hospital builds on a 20-year legacy	3052	265	5	The revived HTx program built on a 20-year history at the University of Mississippi Medical Center	[31]
4	Heart transplant procedure from Montefiore-Einstein, NYC	430,349	1904	1003	HTx with a panel discussion presented by the cardiothoracic surgeons of the Montefiore-Einstein Heart Center on an OR-Live webcast	[32]
5	Surgical Nightmare! Heart Transplant Masterclass - Surgeon Simulator 2013	18,060	3	394	Advertisement (ad) for an electronic game “Surgeon Simulator 2013”	[33]
6	Surgeon Simulator 2013 - Successful heart transplant [Rating: A++]	253,614	3	2469	Ad for an electronic game “Surgeon Simulator 2013”	[34]
7	Heart transplant surgery with live audience	20,086	2	355	Ad for an electronic game “Surgeon Simulator 2013”	[35]
8	Heart transplant part 1	13,676	368	33	Classical and valuable video describing HTx	[36]
9	Implanted heart pumps keep patients in need of transplants alive	240	3	2	KPBS Health Reporter talked about left ventricular-assist device and heart donation	[37]
10	Heart transplant steps simplified by Dr Sandeep Attawar	341	56	2	HTx live broadcast	[38]

^a^The term “relevance” refers to the default ranking system for YouTube queries and is determined based on Google algorithm.

^b^Description states the kind of content present in the videos.

**Table 2:** Detailed characteristics of different categories of YouTube videos with relevant information on HTx.

Characteristics		Useful videos	Misleading videos	Recipients videos	Total HTx-related videos
Number of videos, n (%)		214 (62.6)	6 (1.7)	122 (35.7)	342 (100)
Total duration in minutes (%)		1723.7 (72.61)	26.0 (1.10)	624.1 (26.29)	2373.8 (100)
Mean duration in minutes (SD)		8.05 (13.36)	4.33 (5.33)	5.12 (7.58)	6.94 (11.59)
Mean number of days on YouTube (SD)		582.59 (498.23)	594.17 (519.39)	433.16 (404.05)	529.49 (471.27)
Mean scores (SD), range		16.83 (85.73) 42 to 979	46.50 (110.53) 2 to 272	20.82 (120.47) 0 to 1148	18.77 (99.69) 42 to 1148
Total viewership, n (%)		2,175,845 (56.09)	1,375,673 (35.47)	327,372 (8.44)	3,878,890 (100)
Mean views/day (SD), range		14.31 (53.74) 0.02-523.26	202.07 (324.64) 0.16-785.30	9.69 (38.77) 0.02-359.41	15.95 (67.14) 0.02-785.30
**Source**					
	H/UC^a^	88	0	30	118
	IUC^b^	40	5	45	90
	MDC^c^	22	0	11	33
	NAC^d^	64	1	36	101

^a^Hospital/university channel.

^b^Independent user channel.

^c^Medical dotcom channel.

^d^News agency channel.

**Table 3:** Detailed characteristics of “useful videos” on YouTube uploaded by different sources.

Characteristics	Hospital/university channel	Independent user channel	Medical dotcom channel	News agency channel
Useful videos, N=214 (%)	88 (41.1)	40 (18.7)	22 (10.3)	64 (29.9)
Total duration in minutes, 1723.7 (%)	657.05 (38.12)	320.40 (18.59)	263.00 (15.26)	483.25 (28.03)
Mean duration in minutes (SD)	7.47 (14.04)	8.01 (8.21)	11.95 (21.55)	7.55 (11.35)
Mean number of days on YouTube (SD)	558.67 (456.60)	641.07 (657.85)	412.14 (377.99)	637.53 (456.25)
Mean scores (SD), range	6.70 (34.80) 1 to 327	35.98 (103.36) 1 to 514	6.09 (13.62) 1 to 58	22.47 (126.70) 42 to 979
Total viewership (n) (%)	310,252 (14.26)	1,036,597 (47.64)	88,925 (4.09)	740,071 (34.01)
Mean views/day (SD), range	6.02 (23.82)^a^ 0.07-220.87	39.36 (107.42)^a,b^ 0.02-523.26	10.08 (20.89) 0.08-74.50	11.49 (34.40)^b^ 0.02-224.75

^a^ *P*=.006

^b^ *P*=.046

**Table 4:** Detailed content analysis of “useful videos” from five aspects.

Aspects	Hospital/university channel, n (%)	Independent user channel, n (%)	Medical dotcom channel, n (%)	News agency channel, n (%)	Total, n (%)
Live demonstration of HTx	6 (22.2)	11 (40.7)	5 (18.5)	5 (18.5)	27 (9.4)
Brief introduction of HTx	31 (38.7)	17 (21.2)	18 (22.5)	14 (17.5)	80 (27.9)
Release of new technologies	34 (49.3)	11 (15.9)	6 (8.7)	18 (26.1)	69 (23.9)
Experts’ scholar viewpoints	19 (52.7)	7 (19.5)	5 (13.9)	5 (13.9)	36 (12.5)
Medical humanities	18 (23.7)	17 (22.4)	6 (7.9)	35 (46.1)	76 (26.4)
Coverage on the above five aspects	108 (37.5)	63 (21.9)	40 (13.9)	77 (26.7)	288

## Discussion

### Principal Results

**Figure 3:**
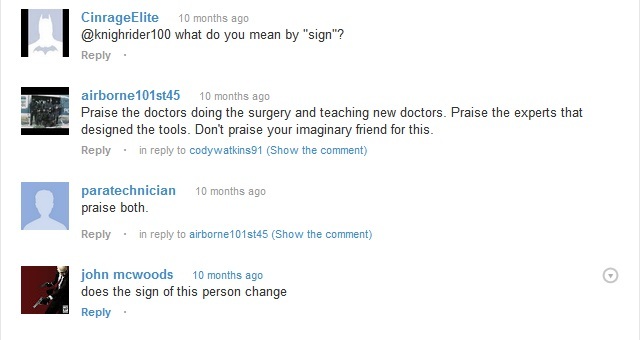
A screenshot of the commendable from Web viewers (on April 12, 2013).

With the rise in popularity of the website YouTube, there is now another route by which medical professionals can learn the specialized knowledge. YouTube enables physicians and institutions with strong reputation to upload multimedia clips of medical knowledge on their channels. For instance, in Table 1, the fourth top video relevant to HTx (see Multimedia Appendix 2) had a high total viewership, which encompassed a live HTx operation with a panel discussion at the Montefiore-Einstein Heart Center, on September 19, 2007. This webcast-featured video portioned the scene of HTx as well as detailed descriptions of surgical techniques. The “scores” (1003 on February 1, 2013) of this video were very high, and it has won a lot of commendableness from physicians and patients, such as “Praise the doctors doing the surgery and teaching new doctors. Praise the experts that designed the tools...” (see Figure 3, comment from a YouTube user named “airborne101st45”).

**Figure 4:**
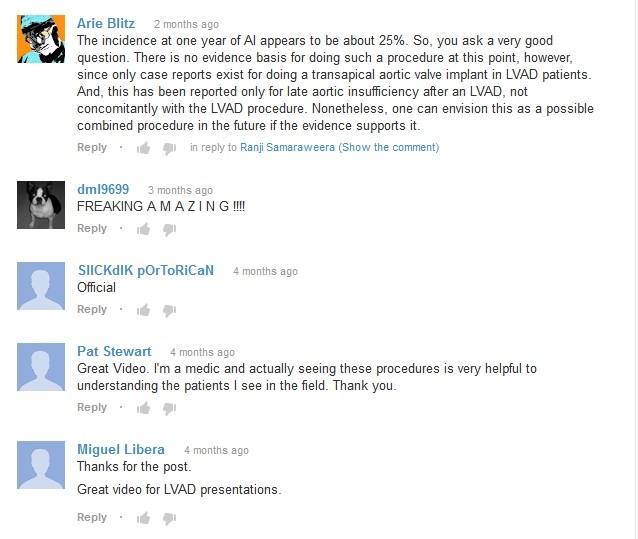
A screenshot of the comments from Web viewers (on April 12, 2013).

**Figure 5:**
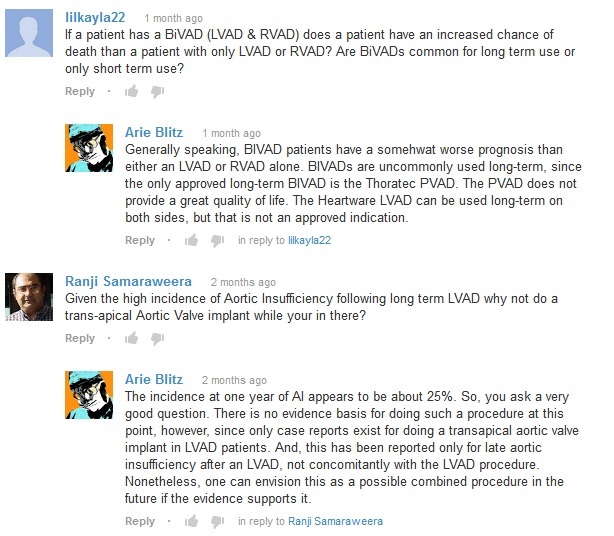
A screenshot of the utterly specialized BiVAD-related discussions (on April 12, 2013).

YouTube videos provide us an effective way to actively engage with our worldwide colleagues, by subscribing and responding to high-quality clips from the respected individual surgeons. Although there is no information available on how many of the viewers of these videos were medical personnel, it is obvious that those high-quality videos can be used by medical staff and can improve the learning outcomes of physicians. For instance, in the live implantation video of a left ventricular assist device (LVAD, Heartmate II) as a bridge to HTx (see Multimedia Appendix 3), Dr Arie Blitz brought clarity and “even made laymen to understand the patients by seeing these procedures” (appraisement from a YouTube user named “Pat Stewart”, see Figure 4). After reviewing utterly specialized questions, such as why not performing a transapical aortic valve combined with LVAD implantation, Dr Arie Blitz always gave counterparts prompt and active responses (Figure 5).

However, our study demonstrated that the majority of HTx-related videos were easily available yet often without expertise information. Considering all the videos that were uploaded before February 1, 2013, YouTube had nearly 7000 videos by searching the keywords about HTx, yet only 342 of the 1800 (19.00%) videos were actually related. Furthermore, in about 40 hours of coverage of these videos, only 72.61% of them contained useful specialized information about this challenging surgery. At present, many reliable academic institutions, such as ISHLT, have not exerted their positive and dominant impacts to increase the signal-to-noise ratio of HTx-related YouTube videos, through uploading their high-quality videos for viewing by people. For example, the American Association for Thoracic Surgery (AATS) has been registered as an organizational user (AATSVideos) on November 7, 2012. However, there were only 21 videos as open resources, which contain nothing about HTx. Interestingly, the striking “Watch AATS Video” sign always stays at the top of AATS website, and there are five links to the Cardiothoracic Surgery Network (CTSNet), which deposited 159 authoritative videos of cardiac surgeries until April 12, 2013. Therefore, even with a newest video titled “Left Ventricular Aneurysm Resection and LVAD Implantation Through Median Sternotomy” (Multimedia Appendix 4) on CTSNet, there was no HTx-related video in AATSVideos Channel. On April 12, 2013, the number of subscribers of “AATSVideos” Channel was still five, and two of the five guys were the authors (X-B Liao and H-M Chen) of this paper. Therefore, with the accumulation of a lot of unrelated videos, there is an exigent need to upload reliable, high-quality HTx-related videos, by professional medical educators, institutions, and organizations.

**Figure 6:**
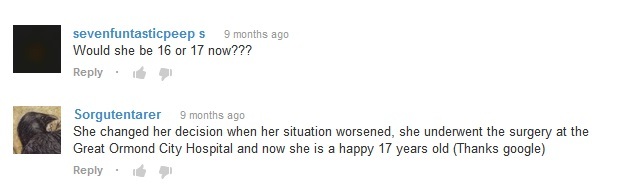
A screenshot of the comments from web viewers about the misleading video (on April 12, 2013).

In addition to the lack of goal-oriented contents from authoritative organizations and trustworthy individuals, there is a real risk of dissemination of misleading information by YouTube. In our study, we found that the maximum-weight misleading video (see Multimedia Appendix 5) accounted for 88.27% (1,214,312/1,375,673) of total viewership, and it arouse a significant support with a “scores” number of 272 (345 “likes”; 73 “dislikes”). The video was uploaded on November 11, 2008 and featured one 13-year-old British girl who claimed to refuse HTx. Her option was obviously irrational and unscientific, thus inevitably, it would negatively affect some viewers. Paradoxically, “She changed her decision when her situation worsened, she underwent the surgery at the Great Ormond City Hospital” (see Figure 6, comment from a YouTube user named “Sorgutentarer”). Nevertheless, this video is still online. Today, content generation is no longer limited to the health care professionals; Web 2.0 services and platforms have empowered patients to create and interact videos with various forms of patient-generated content [40]. Therefore, it is understandable that the emergence of quality-without-assurance videos prompts skepticism and worries along with the obvious information overload.

### Optimizing Directions

Given the problems above, two measures can be implemented by YouTube to improve its functionality with a more efficient way of providing high-quality medical videos. First, a professional community targeting at medical education can be built to inspire specialized videos uploading and consumption, just as iTunes U. Since introduced in 2007, Apple’s iTunes U courses (more than 2500) on a variety of topics have topped a billion downloads, and these courses are contributed by over 2400 educational institutions including universities, colleges, K-12 schools, and districts [40]. As the most advantageous online video provider, YouTube can take use of its incomparably competitive edge to encourage physicians, hospitals, and institutions to upload more instructional videos. Besides, this professional community will bring about specialized ranking outputs by separating e-learning-oriented viewers from common ones just for recreational needs. Based on the specialized community, searching experiences of the users can be optimized and remembered by mining social network characteristics [41], such as an expert assessment model that can strength weight of user rating in ranking factors [42]. Video providers can be scored based on their specialty approbation, and their score can be taken into account and reflected in the ranking system. Moreover, graphical model can be used in social community and medical experts can be distinguished from amateurs [43,44]. Thus more accurate recommendation of relevant videos can be given. Fernandez-Luque et al used a link-based analysis based on a metric called “HealthTrust” to acquire diabetes content from YouTube [45]. Their result indicated that social network analysis could be used to identify trustworthy multimedia in health communities.

Second, more precise and personalized lists of video after searching on YouTube should be provided. When a medical practitioner wants to access HTx-related videos, the most common way is to input keywords (eg, “heart transplantation”), wait for automatic operation embedded in the YouTube search tool, and then look over the videos according to the search result list in sequence. The purposes of viewers for viewing the videos are different, in which some physicians want to look for similar experience as reference for their clinical decision-making or operative skills, and other trainees wish to look up correct and useful information just as learning materials. Though out of different purposes, they don’t expect to be distracted by irrelevant, even misleading information. To meet professional requirements with the least disturbance, YouTube ranking system should be refined and improved.

In the current YouTube ranking system, there are two comprehensive aspects of ranking factors: (1) “content”, which is out of the video self-characteristics, such as correlation between search keywords, video title, and description, and (2) “engagement”, which is contributed by viewer interaction in YouTube social community. There are 12 different detailed factors that are used to determine rank of each video (Table 5) [46]. With the help of these factors, YouTube not only explores the correlation between search keywords and candidate videos but also takes popularity of videos into account. However, the ranking system is mainly based on the relationship of viewership or hits, although YouTube has updated its algorithm with video-discovery features, such as “time watched” [47].

In the present study, it was found that the “scores” of three advertisement videos of “Surgeon Simulator 2013”, an electronic game, were unexpectedly high. However, they were completely uncorrelated with HTx (Table 1). On the other hand, the results of this study showed that IUC videos had significantly higher mean “viewers/day” than H/UC and NAC videos. This phenomenon reflected that viewers are more interested in personal experiences about HTx rather than in professional conferences or didactic lectures, irrespective of authenticity or authority of multimedia materials. Thereupon, this ranking system is still focusing on the popularity of videos, not exactly the accuracy and correlation between videos’ contents and searching keywords.

It is required to take deeper use of social network relationship among viewers. Users’ historical viewing records can be regarded as personal profile, so system can supply personalized search results according to each user’s preference, by using techniques in recommendation system [41], such as collaborating filter and opinion mining [48]. For instance, for a user whose historical viewing records include plenty of cardiac surgery videos, if he/she searches HTx, there is a great possibility that the desired videos are specialized materials for e-learning, other than personal experience sharing just as a recipient. Furthermore, it may benefit the ranking effect to mine literal information generated by viewers via using natural language processing (NLP) techniques, because it can strengthen the correlation between videos and search keywords [49]. So far, superficial literal features on video have been focused on YouTube ranking system; however, there is no evidence that showed that the comments generated by viewers have been noticed enough as they deserved. Most comments contain viewers’ affection response to these videos; therefore, comments can be used to adjust video rating as a feedback. To date, researches on how to efficiently retrieve medical-related videos from YouTube are still few in number. Commonly, collocations in the context are much more informative than frequent phrases [50,51]. Thus, it is probably a suitable means to extract content-related video by NLP-discovered phrases rather than keywords [52,53].

**Table 5:** YouTube ranking factors.^a^

Content	Engagement
Title	Views
Description	Inbound links
Tags	Social shares
Transcriptions	Embeds
Channel authority	Comments
Delivery	Likes and favorites

^a^Reprinted with permission from Chelaru et al [46].

### Study Limitations

First, our study was confined to the content analysis of HTx-related videos retrieved on February 01, 2013. This cross-sectional observation is like a snapshot of information distribution, but the actual source from YouTube is swiftly expanding as one never-ending documentary. Second, our classification method was subjective. However, the kappa coefficient demonstrated fairly high agreement between two cardiac surgeons. We did not extrapolate the percentage of “useful” and “misleading” videos in our dataset to all the HTx-related YouTube videos. Furthermore, our analysis of the comments and the social interactions of viewers and uploaders was based on pure observation without a solid methodological approach. The “scores” are based on “likes” or “dislikes”, and it may not be an excellent indication of viewer preferences or video quality on medical contents. Third, non-English language video clips were excluded, which included some valuable videos, such as many French language HTx-related videos. Fourth, we did not revalidate the results in other networking platforms, such as Baidu or Facebook. Finally, this study was limited to a direct search on YouTube, so we might have missed some valuable surgical videos that could be viewed at other available health information websites.

### Conclusions

This study demonstrates a panoramic view of HTx-related videos on YouTube until February 1, 2013. The results of this study showed that YouTube benefits medical professionals by providing a substantial amount of specialized information. However, casting YouTube to find HTx-related videos is currently inefficient. As more young medical trainees are eagerly participating in social media and e-learning activities, it is reasonable to promote and optimize the dissemination of free and valuable medical videos via YouTube. It is clear from the results of this study that the quality of surgical specialized information in YouTube videos is very heterogeneous and the process of e-learning is not without pitfalls. The reasons include that (1) the content of most videos often lacks institutional or peer quality control, thus the specialized information shared may be not accurate; (2) finding informative and trustworthy targets is hampered by the vast amount of seemingly relevant videos via current ranking system. To solve these problems, we are expecting changes in two aspects. First, more authoritative videos by trusted sources should be posted. Second, ranking system based on present YouTube algorithm may be evolved by adding some elements like peer review, social network analysis, or NLP techniques. With the endeavors of professional individuals, academic institutions, and e-learning communities, YouTube, the leading video streaming websites, will help to meet huge informational needs of medical staffs and promote medical education on HTx.

## Multimedia Appendix 1

The Revived Heart Transplant Program at Batson Children's Hospital builds on a 20-year legacy.

## Multimedia Appendix 2

The heart transplant procedure from Montefiore-Einstein, NYC.

## Multimedia Appendix 3

Example of a high-quality surgical educational video “Heartmate II LVAD Implantation. Arie Blitz, MD” uploaded by “Arie Blitz” channel. It was originally from the YouTube website, reproduced under Creative Commons Attribution License.

## Multimedia Appendix 4

A newest video titled “Left Ventricular Aneurysm Resection and LVAD Implantation Through Median Sternotomy” available on the website of CTSNet; however, it cannot be accessed from “AATSVideos” Channel. It was reproduced under Creative Commons Attribution License.

## Multimedia Appendix 5

The maximum-weight misleading video “13-year-old British girl” uploaded by “uniquerocks” Channel. It was from originally from the YouTube website, reproduced under Creative Commons Attribution License.

## Abbreviations

AATS: American Association of Thoracic Surgery
ANOVA: one-way analysis of variance
CTSNet: Cardiothoracic Surgery Network
HTx: heart transplantation
H/UC: hospital/university channel
ISHLT: International Society for Heart and Lung Transplantation
IUC: independent user channel
LVAD: left ventricular assist device
MDC: medical dotcom channel
NAC: news agency channel
NLP: natural language processing
